# Maternal expressions of warmth and negativity and adolescent mental health: using longitudinal monozygotic twin‐difference analyses to approach causal inference

**DOI:** 10.1111/jcpp.70020

**Published:** 2025-08-13

**Authors:** Alice Wickersham, Avshalom Caspi, Louise Arseneault, Terrie E. Moffitt, Johnny Downs, Antony Ambler, Rachel M. Latham, Nicholas Cummins, Zoë Firth, Jasmin Wertz, Helen L. Fisher

**Affiliations:** ^1^ CAMHS Digital Lab, Department of Child and Adolescent Psychiatry Institute of Psychiatry, Psychology & Neuroscience, King's College London London UK; ^2^ Social Genetic and Developmental Psychiatry Centre, Institute of Psychiatry, Psychology & Neuroscience, King's College London London UK; ^3^ Department of Psychology and Neuroscience Duke University Durham NC USA; ^4^ Duke University Population Research Institute, Duke University Durham NC USA; ^5^ Department of Psychiatry and Behavioral Sciences Duke University Durham NC USA; ^6^ PROMENTA, Department of Psychology, University of Oslo Oslo Norway; ^7^ South London and Maudsley NHS Foundation Trust London UK; ^8^ ESRC Centre for Society and Mental Health King's College London London UK; ^9^ Department of Biostatistics and Health Informatics Institute of Psychiatry, Psychology & Neuroscience, King's College London London UK; ^10^ Thymia London UK; ^11^ Department of Psychology University of Edinburgh Edinburgh UK

**Keywords:** Expressed emotion, mental health, adolescence, cohort study, parenting, twins

## Abstract

**Background:**

Emotions that mothers express about their offspring are associated with offspring mental health during childhood, but little research has explored whether this extends into adolescence. We investigated associations between maternal warmth and negativity towards twin offspring at age 10, and subsequent mental health outcomes in early and late adolescence.

**Methods:**

The Environmental Risk (E‐Risk) Longitudinal Twin Study is a population‐based cohort of 2,232 same‐sex twins born in 1994–1995 across England and Wales. Maternal warmth and negativity were assessed from Five‐Minute Speech Samples obtained when twins were aged 10. Continuous mental health outcomes were assessed in interviews with twins at ages 12 (depression, anxiety, attention‐deficit hyperactivity disorder and conduct disorder) and 18 (general psychopathology, internalising, externalising and thought disorder). Linear regressions were conducted on 1906 participants with available data and adjusted for sex, family socioeconomic status, and age‐5 emotional and behavioural problems. We then conducted a monozygotic twin‐difference analysis to control for unmeasured shared environmental and genetic factors.

**Results:**

Lower maternal warmth and higher maternal negativity were associated with worse mental health outcomes at ages 12 and 18. For example, when comparing differences in mothers' expressed emotions and mental health outcomes within monozygotic twin pairs, higher negativity remained associated with externalising symptoms (*b* = 1.77, 95% CI = 0.68 to 2.86, *β* = .14) and poorer general psychopathology (*b* = 1.82, 95% CI = 0.63 to 3.01, *β* = .13), and lower warmth with externalising symptoms (*b* = −1.96, 95% CI = −3.54 to −0.37, *β* = −.11). These associations remained after adjusting for twin‐differences in age‐5 emotional and behavioural problems and birth weight. Null findings were more frequently observed for maternal warmth and internalising outcomes.

**Conclusions:**

Using a genetically sensitive design to approach causal inference, we found evidence for associations between maternal warmth/negativity and adolescent mental health outcomes. Maternal expressed emotion ratings might provide an early opportunity to identify families who would benefit from interventions and mental health disorder prevention programmes.

## Introduction

Approximately one in eight children and adolescents in England have a mental health disorder (Sadler et al., 2018). Poor mental health during this period is associated with poorer longitudinal outcomes across various domains, including worse school, social, economic and health outcomes (Clayborne, Varin, & Colman, 2019). Identifying adolescents at increased risk of mental health difficulties as early as possible is needed to ensure that preventative interventions can be provided to those who are most vulnerable to mitigate the development of these often chronic and disabling disorders (Caspi et al., 2020).

One potential early indicator of risk is expressed emotion (EE) within the family environment. EE refers to the type and intensity of emotions shown by caregivers towards family members (Rutter & Brown, 1966), thereby offering a well‐established index of the home's emotional environment. Over several decades, a wide range of coding frameworks have been developed to measure EE, many based on speech elicited by caregivers about their family members through the Five‐Minute Speech Sample (FMSS) (Magaña et al., 1986; Sher‐Censor, 2015). These frameworks variously measure thoughts and feelings such as criticism, emotional over‐involvement and hostility, among others. Two global scales of EE, which have been widely used in studies of parents and young offspring and are the focus of this study, are warmth (capturing affection, empathy, enthusiasm and interest) and negativity (capturing criticism, resentment and hostility) (Caspi et al., 2004).

EE, as measured using various frameworks, has been shown to be a robust predictor of poor outcomes in the adult mental health literature. For example, high levels of negatively expressed emotions from relatives predicted greater likelihood of relapses among adults with psychiatric disorders (Amaresha & Venkatasubramanian, 2012; Hooley, 2007). More recently, the focus of EE research has shifted towards prediction of child and adolescent mental health (Peris & Miklowitz, 2015). It has also been argued that EE within families might play a particularly powerful role in child and adolescent wellbeing (Vaughn, 1989). For example, under a ‘toxic family stress’ model of EE (Peris & Miklowitz, 2015), growing up with frequent criticism in a ‘high EE’ household could present a persistent stressor that creates and exacerbates poor mental health and interacts with biological vulnerabilities to mental illness.

Studies have shown that ratings of negative emotions from parents' speech are associated with mental health issues in children including anxiety (Gar & Hudson, 2008), depression (Schwartz, Dorer, Beardslee, Lavori, & Keller, 1990) and attention‐deficit hyperactivity disorder (ADHD) (Peris & Hinshaw, 2003). However, without longitudinal studies that can take account of possible genetic and environmental confounding, it is not possible to infer a potential causal relationship. Genetics could influence both maternal EE and the onset of mental health problems, necessitating designs which can rule out this confounder to demonstrate the importance of nonshared environmental risk factors like parental attitudes (which can vary both within and between families). Accordingly, a previous study conducted a monozygotic (MZ) twin‐difference analysis to investigate the association between differences in maternal EE for twins within a pair and subsequent differences in antisocial behavioural problems for these twins (Caspi et al., 2004). Their genetically sensitive design enabled them to control for unmeasured confounding in the shared family environment (as twins were brought up in the same households) and genetic confounding (as MZ twins share all of their genes). Their findings showed that lower maternal warmth and higher maternal negativity, as rated from speech samples at age 5, may play a causal role in the development of greater antisocial behavioural problems at age 7.

Given that adolescence is a key period for the emergence of mental health issues, with approximately one third of adults having experienced mental disorder onset by age 15, and two thirds by age 18 (Caspi et al., 2020), it is important to identify factors that are associated with the onset of mental health issues in this developmental period. However, there has been little research exploring whether EE is associated with mental health issues during adolescence. Additionally, as mental health disorders often co‐occur and symptoms tend to overlap between diagnostic categories (Caspi et al., 2020; Kotov et al., 2017), it is important to focus on dimensional measures of mental health in adolescence and overarching measures that capture multiple aspects of psychopathology (Caspi, Houts, Fisher, Danese, & Moffitt, 2023). From a clinical perspective, understanding these issues could provide an opportunity to identify families who may benefit most from family or parenting interventions to facilitate early prevention of mental health disorders in children.

The objective of this study was to investigate the association between mothers' warmth and negativity towards their twin offspring measured at age 10, and the mental health outcomes of these twins assessed in early (age 12) and late (age 18) adolescence in the Environmental Risk (E‐Risk) Longitudinal Twin Study. While previous studies show that fathers and other caregivers also play an important role, mothers were predominantly identified as the ‘primary caregivers’ by families participating in E‐Risk, and were therefore the focus of the interviews used in this study. In accordance with a previous study by Caspi et al. (2004), we hypothesise that higher maternal negativity and lower warmth at age 10 will be associated with higher scores on all measures of mental ill‐health at ages 12 and 18 years (given the multitude of mental health outcomes previously associated with EE). In order to get closer to causal inference, we will use MZ twin‐difference analyses (Caspi et al., 2004) to examine associations between differences in age‐10 warmth and negativity ratings between MZ twins in a pair and differences in their scores on continuous mental health measures at ages 12 and 18.

## Methods

Reporting follows Strengthening the Reporting of Observational Studies in Epidemiology (STROBE) guidelines for cohort studies (Table S1) (Vandenbroucke et al., 2007).

### E‐Risk Study Cohort

Participants were members of the E‐Risk Study, which tracks the development of a nationally representative birth cohort of 2,232 British twin children. The sample was drawn from a larger birth register of twins born in England and Wales in 1994–1995 (Trouton, Spinath, & Plomin, 2002). Full details about the sample are reported elsewhere (Moffitt & E‐Risk Study Team, 2002). Briefly, the E‐Risk sample was constructed in 1999–2000, when 1,116 families (93% of those eligible) with same‐sex 5‐year‐old twins participated in home‐visit assessments. This sample comprised 56% MZ and 44% dizygotic (DZ) twin pairs; sex was evenly distributed within zygosity (49% male). Families were recruited to represent the UK population of families with newborns in the 1990s, on the basis of residential location throughout England and Wales and mother's age. Teenaged mothers with twins were overselected to replace high‐risk families who were selectively lost to the register through nonresponse. Older mothers having twins via assisted reproduction were underselected to avoid an excess of well‐educated older mothers.

This sampling strategy led to the study sample representing the full range of socioeconomic conditions in Great Britain, as reflected in the families' distribution on a neighbourhood‐level socioeconomic index [ACORN (A Classification of Residential Neighbourhoods) developed by CACI Inc. for commercial use; (CACI Information Services, 2006)]. E‐Risk families' ACORN distribution closely matches that of households nationwide: 25.6% of E‐Risk families live in ‘wealthy achiever’ neighbourhoods compared to 25.3% of households nationwide; 5.3% versus 11.6% live in ‘urban prosperity’ neighbourhoods; 29.6% versus 26.9% live in ‘comfortably off’ neighbourhoods; 13.4% versus 13.9% live in ‘moderate means’ neighbourhoods; and 26.1% versus 20.7% live in ‘hard‐pressed’ neighbourhoods. E‐Risk underrepresents urban prosperity neighbourhoods because such households are likely to be childless. Further details about the E‐Risk sample, including parental characteristics, home and neighbourhood socioeconomic details, and family structure are reported by Moffitt and E‐Risk Study Team (2002).

Follow‐up home‐visits were conducted when children were aged 7, 10, 12 and 18 years (participation rates were 98%, 96%, 96% and 93%, respectively). Home‐visits at ages 5, 7, 10 and 12 years included assessments with participants as well as their mother (or primary caregiver); the home‐visit at age 18 included interviews only with the participants. Each participant in a twin pair was assessed by a different interviewer. There were 2,066 E‐Risk participants who were assessed at age 18. The average age of the participants at the time of the assessment was 18.4 years (*SD* = 0.36); all interviews were conducted after the 18th birthday. There were no differences between those who did and did not take part at age 18 in terms of socioeconomic status (SES) assessed when the cohort was initially defined (χ^2^ = 0.86, *p* = .65), age‐5 IQ scores (*t* = 0.98, *p* = .33), age‐5 internalising or externalising behaviour problems (*t* = 0.40, *p* = .69 and *t* = 0.41, *p* = .68, respectively) or childhood poly‐victimisation (*z* = 0.51, *p* = .61).

The Joint South London and Maudsley and the Institute of Psychiatry Research Ethics Committee approved each phase of the study (NRES 1997/122). Parents gave informed consent and twins gave assent between 5 and 12 years and then informed consent at age 18.

### Measures

#### Maternal expressed emotion, age 10

Maternal EE was measured as previously described elsewhere (Caspi et al., 2004). Briefly, we used a 5‐min speech sample to elicit EE about each child. Trained interviewers asked caregivers, when the twins were aged 10, to describe each of their children (‘For the next 5 minutes, I would like you to describe [child] to me; what is [child] like?’). The mother was encouraged to talk freely with few interruptions. However, if the mother found this difficult, the interviewer could aid the mother with a series of semi‐structured probes, such as ‘In what ways would you like [child] to be different?’ Interviews about each twin were separated in time by approximately 90 min. All interviews were audiotaped with the mother's consent. Data for EE were missing for 6% of the sample because some mothers did not wish to be audiotaped, because of technical problems with the tape or because of loss to follow‐up.

Two trained raters coded the audiotapes according to guidelines adapted from the FMSS scoring manual (Magaña et al., 1986), which were modified for use with preschool children (Caspi et al., 2004; see also Daley, Sonuga‐Barke, & Thompson, 2003; Sandberg, Rutter, & Järvi, 2003). The raters underwent 2 weeks of training about coding EE. Interrater reliability was established by having the raters individually code audiotapes describing 40 children (ranging from *r* = .84 for negativity to *r* = .90 for warmth). The same rater coded both twins in the same family. The rater was blind to all other E‐Risk Study data. We examined two variables coded from the FMSS: warmth and negativity. Warmth captured expressions such as sympathy, empathy, enthusiasm, interest and enjoyment in the child, while negativity captured expressions such as dissatisfaction, criticism, disparagement and hostility. Warmth and negativity were global measures used to describe the whole speech sample and were each coded on 6‐point Likert ordinal scales (Table 1) based on both what was said and the tone of voice used. Details of the scoring framework used in this study are provided in Appendix S1, and further information can be found in Caspi et al. (2004). It should be noted that warmth and negativity do not reflect opposites of a single spectrum (e.g. it is possible to score low on both warmth and negativity). The correlation between negativity and warmth ratings at age 10 was *r* = −.50 (*p* < .001, *n* = 2,099). Additional information about the measurement, reliability and concurrent validity of maternal EE is reviewed by Sandberg et al. (2003).

**Table 1:** Summary of warmth and negativity rating scales

Score	Warmth	Negativity
0	*No warmth*	*No negativity*
1	*Very little warmth* – only a slight amount of understanding, sympathy, concern, enthusiasm about or interest in the child	*A little negativity* – one minor criticism of the child
2	*Some warmth* – a detached, rather clinical approach and little or no warmth of tone, but moderate understanding	*Some negativity* – two criticisms of the child that were stronger in tone
3	*Moderate warmth* – definite understanding, sympathy and concern, but only limited warmth of tone	*Moderate negativity* – some dissatisfaction; repeatedly mentions 1–2 particular traits of the child that she did not like and wished to change
4	*Moderately high warmth* – more tonal warmth is evident along with concern about the child and some enthusiasm, interest in and/or enjoyment of the child	*Moderately high negativity* – makes disparaging remarks and finds fault with the child
5	*High warmth* – definite and clear‐cut tonal warmth, enthusiasm, interest in and enjoyment of the child	*Resentful and hostile* – actively dislikes the child

These are global measures used to describe the whole speech sample based on both what was said and the tone of voice used (see Caspi et al., 2004 for full details). For negativity, codes 3–5 were considered present when maternal negativity was generalised to the child himself or herself rather than against particular behaviours or attributes. These ratings were used when the tone and content of the interview were primarily negative.

#### Adolescent mental health outcomes, age 12

Symptoms of depression and anxiety were assessed using the Children's Depression Inventory (CDI) (Kovacs, 1992) and the Multidimensional Anxiety Scale for Children (MASC) (March & Parker, 2014), respectively, in private interviews with each twin. Conduct disorder symptoms over the preceding 6 months were based on twins' self‐report using DSM‐IV diagnostic criteria (American Psychiatric Association, 1994). ADHD symptoms for the preceding 6 months were ascertained by mothers' and teachers' reports of inattention, impulsivity and hyperactivity derived from DSM‐IV diagnostic criteria (American Psychiatric Association, 1994) and the Rutter Child Scales (Sclare, 1997). We formed an ADHD symptom scale by summing the mother and teacher ratings to obtain a highly reliable overall measure (Kuntsi et al., 2004).

#### Adolescent mental health outcomes, age 18

Participants were interviewed about past‐year symptoms of mental disorder. These methods have been previously described (Reuben et al., 2021; Schaefer et al., 2018) and are fully detailed in Appendix S2. Briefly, five externalising‐spectrum disorder symptoms were assessed, including alcohol dependence, cannabis dependence, tobacco dependence, conduct disorder and ADHD. Four internalising‐spectrum disorder symptoms were assessed, including depression, generalised anxiety disorder, posttraumatic stress disorder and eating disorder. Thought disorder symptoms were assessed via seven items about delusions and hallucinations and six items about unusual thoughts and feelings. For analysis, we used factor scores that had been derived from these symptoms as part of a previous study (Schaefer et al., 2018): an internalising factor, an externalising factor, a thought disorder factor and a general psychopathology factor, or ‘p‐factor’. Details of the confirmatory factor analysis underlying these variables are provided in the original paper (Schaefer et al., 2018) and summarised with further details in Appendix S2 and Figure S1. For expository purposes, scores on each factor were scaled to a mean (*SD*) of 100 (15).

### Covariates

Variables used as covariates included biological sex and weight at birth, and family SES at age 5. Birth weight (in g) was used as a proxy for potential neurological differences between twins in a pair that may result in differential attitudes by the mother towards them (Caspi et al., 2004), as well as potentially influencing differences in their mental health outcomes (Pettersson, Larsson, D'Onofrio, Almqvist, & Lichtenstein, 2019). Family SES was measured via a composite of parental income, education and occupation measured when participants were aged 5. The three SES indicators were highly correlated (*r*'s ranged from .57 to .67, *p* < .05) and loaded significantly onto one latent factor (factor loadings = 0.80, 0.70 and 0.83 for income, education and occupation, respectively). The latent variable was categorised into tertiles (i.e. low‐, medium‐ and high‐SES) (Trzesniewski, Moffitt, Caspi, Taylor, & Maughan, 2006).

Childhood emotional and behavioural problems occurring prior to the EE assessment were also considered as covariates to account for pre‐existing mental health issues and reduce the possibility of reverse causation. These were assessed when the twins were aged 5 using the Child Behaviour Checklist in interviews with mothers and the Teacher Report Form by post for teachers (Achenbach, 1991a, 1991b). The emotional (internalising) problems scale is the sum of items in the withdrawn and anxious/depressed subscales, and the behavioural (externalising) problems scale is the sum of items from the aggressive and delinquent behaviour subscales. The internal consistencies of mothers' and teachers' reports were .88 and .93, respectively. We summed and standardised mothers' and teachers' reports of each of these measures to create cross‐informant scales representing total emotional and behavioural problems.

### Statistical analysis

Our first set of analyses investigated whether maternal warmth and negativity at age 10 was associated with adolescent mental health at ages 12 and 18, comparing children in different families. Linear regressions were conducted to test associations between (i) maternal negativity and (ii) maternal warmth and each adolescent mental health outcome variable in turn. These models were first unadjusted, then adjusted for biological sex and family SES and then additionally adjusted for emotional and behavioural problems at age 5. Family‐level clustering was accounted for using cluster‐robust standard errors (StataCorp, 2023).

Our second set of analyses investigated how similar MZ twins were in their maternal warmth and negativity ratings and in their adolescent mental health outcomes. Pearson intrapair correlations were conducted between twins on these variables after limiting the sample to MZ twins.

Our final set of analyses investigated whether differences in maternal warmth and negativity were related to differences in adolescent mental health for MZ twins reared in the same family. We calculated MZ twin‐difference scores in maternal warmth and negativity ratings and in adolescent mental health scores. We then conducted linear regressions investigating associations between MZ twin‐differences in (i) maternal negativity and (ii) maternal warmth and MZ twin‐differences in each adolescent mental health outcome. These regressions were first unadjusted, then adjusted for twin‐differences in emotional and behavioural problems at age 5, then additionally adjusted for twin‐differences in birth weight. All analyses were conducted in Stata version 17 (StataCorp, 2023), and the statistical significance threshold was set at *p* < .05.

### Missing data

Of the 2,232 twin children in the E‐Risk study cohort at age 5, a total of 1,906 (85.4%) participants had complete data for both twins on maternal EE at age 10 and all adolescent mental health outcomes at age 12 and age 18 (Table S2). The characteristics of participants with complete data on these variables were very similar to the characteristics of the full cohort (Table S3), so we conducted complete case analyses. Of the 529 MZ twin pairs included in our twin‐difference analysis, 40 participants were additionally missing the birth weight covariate. This was again handled using complete case analyses, such that 489 twin pairs were included in analyses that adjusted for twin differences in birth weight.

## Results

Participant characteristics are described in Table 2. There were slightly more girls (53.2%) than boys (46.8%) and identical (55.5%) than nonidentical (44.5%) twins in the analysis sample. The majority were Caucasian (90.5%), and families were split evenly across socioeconomic strata.

**Table 2:** Sample characteristics and variables used in this analysis, *n* = 1,906

	Frequency
	*n* (%)
Biological sex at birth	
Male	892 (46.8)
Female	1,014 (53.2)
Zygosity	
Monozygotic	1,058 (55.5)
Dizygotic	848 (44.5)
Family socioeconomic status, age 5	
Low	636 (33.4)
Medium	622 (32.6)
High	648 (34.0)
Ethnicity	
White	1,724 (90.5)
Asian	78 (4.1)
Black	34 (1.8)
Mixed race	6 (0.3)
Other	64 (3.4)

	Mean (*SD*)
Birth weight (g)	2446.7 (536.2)
Maternal expressed emotion, age 10	
Warmth	3.7 (0.9)
Negativity	1.4 (0.9)
Mental health, age 5	
Behavioural problems	18.0 (13.7)
Emotional problems	12.0 (8.1)
Mental health, age 12	
Depression symptoms	3.1 (5.3)
Anxiety symptoms	7.6 (3.0)
ADHD symptoms	12.1 (11.1)
Conduct disorder symptoms	1.0 (1.7)
Mental health, age 18	
General Psychopathology (P‐factor)	99.8 (15.0)
Internalising symptoms	99.8 (15.0)
Externalising symptoms	99.7 (15.0)
Thought disorder symptoms	99.8 (14.9)

Birth weight was missing for *n* = 142. ADHD, attention deficit hyperactivity disorder; *SD*, standard deviation.

### Analysis 1: Is childhood maternal EE associated with adolescent mental health? Comparing adolescents across different families

Unadjusted analyses indicated that higher maternal warmth at age 10 was associated with lower scores on all adolescent mental health outcomes (Table 3). However, following adjustment for biological sex, family SES, and prior behavioural and emotional problems, these associations only remained statistically significant for depression (age 12; adjusted *b* = −0.69, 95% CI = −0.99 to −0.38, *β* = −.12), ADHD (age 12; adjusted *b* = −1.30, 95% CI = −1.85 to −0.75, *β* = −.10), conduct disorder (age 12; adjusted *b* = −0.33, 95% CI = −0.44 to −0.23, *β* = −.18) and externalising symptoms (age 18; adjusted *b* = −1.46, 95% CI = −2.35 to −0.57, *β* = −.09).

**Table 3:** Associations between childhood maternal expressed emotion and each adolescent mental health outcome

Mental health outcome	Maternal warmth exposure, age 10								
	Model 1			Model 2			Model 3		
	*b* (95% CI)	*p*	*β*	*b* (95% CI)	*p*	*β*	*b* (95% CI)	*p*	*β*
Age 12									
Depression symptoms	−0.87 (−1.19 to −0.54)	<.001	−.15	−0.81 (−1.13 to −0.49)	<.001	−.14	−0.69 (−0.99 to −0.38)	<.001	−.12
Anxiety symptoms	−0.21 (−0.39 to −0.04)	.016	−.06	−0.17 (−0.34 to 0.00)	.054	−.05	−0.14 (−0.31 to 0.03)	.106	−.04
ADHD symptoms	−2.60 (−3.27 to −1.93)	<.001	−.21	−1.96 (−2.58 to −1.34)	<.001	−.16	−1.30 (−1.85 to −0.75)	<.001	−.10
Conduct disorder symptoms	−0.50 (−0.62 to −0.38)	<.001	−.27	−0.43 (−0.54 to −0.31)	<.001	−.23	−0.33 (−0.44 to −0.23)	<.001	−.18
Age 18									
General psychopathology (P‐factor)	−1.78 (−2.62 to −0.94)	<.001	−.11	−1.25 (−2.09 to −0.40)	.004	−.07	−0.82 (−1.65 to 0.01)	.054	−.05
Internalising symptoms	−1.35 (−2.19 to −0.52)	.002	−.08	−0.91 (−1.76 to −0.07)	.035	−.05	−0.52 (−1.35 to 0.32)	.226	−.03
Externalising symptoms	−2.56 (−3.46 to −1.65)	<.001	−.15	−1.94 (−2.85 to −1.02)	<.001	−.11	−1.46 (−2.35 to −0.57)	.001	−.09
Thought disorder symptoms	−1.68 (−2.50 to −0.86)	<.001	−.10	−1.17 (−1.98 to −0.35)	.005	−.07	−0.77 (−1.58 to 0.03)	.060	−.05

Mental health outcome	Maternal negativity exposure, age 10								
	Model 1			Model 2			Model 3		
	*b* (95% CI)	*p*	*β*	*b* (95% CI)	*p*	*β*	*b* (95% CI)	*p*	*β*
Age 12									
Depression symptoms	0.96 (0.66 to 1.27)	<.001	.17	0.92 (0.63 to 1.22)	<.001	.16	0.75 (0.45 to 1.05)	<.001	.13
Anxiety symptoms	0.16 (0.01 to 0.31)	.041	.05	0.16 (0.01 to 0.31)	.042	.05	0.13 (−0.02 to 0.28)	.094	.04
ADHD symptoms	3.88 (3.26 to 4.50)	<.001	.33	3.35 (2.78 to 3.91)	<.001	.28	2.23 (1.70 to 2.75)	<.001	.19
Conduct disorder symptoms	0.59 (0.47 to 0.70)	<.001	.33	0.53 (0.42 to 0.63)	<.001	.30	0.37 (0.27 to 0.46)	<.001	.21
Age 18									
General psychopathology (P‐factor)	2.54 (1.76 to 3.33)	<.001	.16	2.28 (1.48 to 3.07)	<.001	.14	1.56 (0.78 to 2.35)	<.001	.10
Internalising symptoms	2.01 (1.24 to 2.78)	<.001	.13	1.85 (1.07 to 2.63)	<.001	.12	1.20 (0.41 to 1.98)	.003	.07
Externalising symptoms	3.32 (2.53 to 4.11)	<.001	.21	2.85 (2.07 to 3.64)	<.001	.18	2.00 (1.23 to 2.78)	<.001	.12
Thought disorder symptoms	2.46 (1.66 to 3.26)	<.001	.15	2.19 (1.39 to 3.00)	<.001	.14	1.57 (0.78 to 2.36)	<.001	.10

*n* = 1,906 individuals. Standard errors have been adjusted for family‐level clustering. Model 1: Unadjusted, univariable linear regressions. Model 2: Multivariable linear regressions adjusted for biological sex and family socioeconomic status. Model 3: Multivariable linear regressions adjusted for biological sex, family socioeconomic status, behavioural problems at age 5 and emotional problems at age 5. ADHD, attention deficit hyperactivity disorder; *b*, unstandardised regression coefficient; CI, confidence interval; *β*, standardised regression coefficient (note these have been calculated without taking into account clustering).

Additionally, higher maternal negativity was associated with higher symptom scores on all adolescent mental health outcomes (Table 3). These associations all remained statistically significant following adjustment for covariates, except for anxiety (age 12).

### Analysis 2: How similar or different are MZ twins on their childhood maternal EE ratings and their adolescent mental health outcomes?

Table 4 shows the correlations between MZ twins in their age‐10 maternal EE exposures and adolescent mental health outcomes. MZ twins were only moderately correlated in their maternal EE ratings, with greater similarities in their warmth ratings (*r* = .65) than in their negativity ratings (*r* = .31). Intrapair correlations on adolescent mental health outcomes ranged from weak [*r* = .28, depression (age 12)] to moderate [*r* = .69, ADHD and conduct disorder (age 12)]. Since these twins are genetically identical, this suggests that between one third and three quarters of the variance in these outcomes could be attributed to nonshared environmental factors (plus measurement error) (1–0.69 = 0.31; 1–0.28 = 0.72).

**Table 4:** Intrapair correlations showing similarities between monozygotic twins in their childhood maternal expressed emotion exposure and adolescent mental health outcomes

	Pearson correlation coefficient, *p* value
Maternal expressed emotion, age 10	
Warmth	*r* = .65, *p* < .001
Negativity	*r* = .31, *p* < .001
Mental health, age 12	
Depression symptoms	*r* = .28, *p* < .001
Anxiety symptoms	*r* = .40, *p* < .001
ADHD symptoms	*r* = .69, *p* < .001
Conduct disorder symptoms	*r* = .69, *p* < .001
Mental health, age 18	
General psychopathology (P‐factor)	*r* = .52, *p* < .001
Internalising symptoms	*r* = .52, *p* < .001
Externalising symptoms	*r* = .60, *p* < .001
Thought disorder symptoms	*r* = .45, *p* < .001

*n* = 529 monozygotic twin pairs. ADHD, attention deficit hyperactivity disorder.

### Analysis 3: Are differences in childhood maternal EE related to differences in adolescent mental health outcomes for MZ twins reared in the same family?

Table 5 shows the associations between MZ twin differences in maternal EE at age 10 and MZ twin differences in adolescent mental health outcomes (MZ twin‐pair is the unit of analysis). On average, the twin experiencing greater maternal warmth went on to experience lower levels of ADHD (age 12; unadjusted *b* = −1.39, 95% CI = −2.43 to −0.36, *β* = −.11), conduct disorder (age 12; unadjusted *b* = −0.28, 95% CI = −0.43 to −0.13, *β* = −.16) and externalising symptoms (age 18; unadjusted *b* = −1.96, 95% CI = −3.54 to −0.37, *β* = −.11) compared to their genetically identical cotwin, although the association with ADHD symptoms was no longer statistically significant after adjusting for prior emotional and behavioural problems and birth weight.

**Table 5:** Associations between monozygotic (MZ) twin‐differences in childhood maternal expressed emotion and MZ twin‐differences in each adolescent mental health outcome

MZ twin‐differences in outcome	MZ twin‐differences in maternal warmth exposure, age 10								
	Model 1			Model 2			Model 3		
	*b* (95% CI)	*p*	*β*	*b* (95% CI)	*p*	*β*	*b* (95% CI)	*p*	*β*
Age 12									
Depression symptoms	−0.39 (−1.07 to 0.30)	.266	−.05	−0.38 (−1.06 to 0.31)	.281	−.05	−0.32 (−1.01 to 0.37)	.368	−.04
Anxiety symptoms	−0.21 (−0.59 to 0.16)	.259	−.05	−0.25 (−0.62 to 0.13)	.194	−.06	−0.28 (−0.66 to 0.10)	.153	−.06
ADHD symptoms	−1.39 (−2.43 to −0.36)	.009	−.11	−1.15 (−2.17 to −0.13)	.028	−.09	−1.05 (−2.11 to −0.00)	.050	−.09
Conduct disorder symptoms	−0.28 (−0.43 to −0.13)	<.001	−.16	−0.26 (−0.41 to −0.11)	.001	−.14	−0.24 (−0.39 to −0.09)	.002	−.14
Age 18									
General psychopathology (P‐factor)	−1.56 (−3.28 to 0.17)	.078	−.08	−1.46 (−3.20 to 0.28)	.099	−.07	−1.47 (−3.22 to 0.27)	.097	−.08
Internalising symptoms	−1.23 (−2.97 to 0.51)	.166	−.06	−1.14 (−2.90 to 0.61)	.201	−.06	−1.20 (−2.96 to 0.55)	.178	−.06
Externalising symptoms	−1.96 (−3.54 to −0.37)	.015	−.11	−1.80 (−3.39 to −0.22)	.026	−.10	−1.69 (−3.31 to −0.06)	.042	−.09
Thought disorder symptoms	−1.54 (−3.37 to 0.29)	.100	−.07	−1.47 (−3.31 to 0.37)	.118	−.07	−1.48 (−3.33 to 0.37)	.117	−.07

MZ twin‐differences in outcome	MZ twin‐differences in maternal negativity exposure, age 10								
	Model 1			Model 2			Model 3		
	*b* (95% CI)	*p*	*β*	*b* (95% CI)	*p*	*β*	*b* (95% CI)	*p*	*β*
Age 12									
Depression symptoms	0.47 (−0.00 to 0.94)	.052	.08	0.47 (−0.01 to 0.94)	.056	.08	0.40 (−0.09 to 0.89)	.106	.07
Anxiety symptoms	0.09 (−0.17 to 0.35)	.483	.03	0.13 (−0.13 to 0.39)	.316	.04	0.17 (−0.10 to 0.44)	.214	.06
ADHD symptoms	1.66 (0.96 to 2.37)	<.001	.20	1.40 (0.70 to 2.11)	<.001	.17	1.42 (0.69 to 2.15)	<.001	.17
Conduct disorder symptoms	0.21 (0.11 to 0.32)	<.001	.17	0.19 (0.08 to 0.29)	.001	.15	0.17 (0.06 to 0.27)	.003	.14
Age 18									
General psychopathology (P‐factor)	1.82 (0.63 to 3.01)	.003	.13	1.74 (0.54 to 2.95)	.005	.12	1.57 (0.34 to 2.79)	.012	.12
Internalising symptoms	1.49 (0.29 to 2.69)	.015	.11	1.42 (0.20 to 2.64)	.023	.10	1.18 (−0.06 to 2.41)	.062	.09
Externalising symptoms	1.77 (0.68 to 2.86)	.001	.14	1.61 (0.51 to 2.72)	.004	.13	1.52 (0.38 to 2.66)	.009	.12
Thought disorder symptoms	1.94 (0.68 to 3.20)	.003	.13	1.89 (0.61 to 3.17)	.004	.13	1.77 (0.47 to 3.07)	.008	.12

Models 1 and 2 comprise *n* = 529 MZ twin pairs. Model 3 comprises *n* = 489 MZ twin pairs with available birth weight data. Model 1: Unadjusted, univariable linear regressions. Model 2: Multivariable linear regressions adjusted for MZ twin‐differences in behavioural problems at age 5, and emotional problems at age 5. Model 3: Multivariable linear regressions adjusted for MZ twin‐differences in behavioural problems at age 5, emotional problems at age 5 and birth weight. ADHD, attention deficit hyperactivity disorder; *b*, unstandardised regression coefficient; CI, confidence interval; MZ, monozygotic; *β*, standardised regression coefficient.

Additionally, on average, the twin experiencing greater maternal negativity went on to experience higher levels of ADHD (age 12; unadjusted *b* = 1.66, 95% CI = 0.96 to 2.37, *β* = .20), conduct disorder (age 12; unadjusted *b* = 0.21, 95% CI = 0.11 to 0.32, *β* = .17) and all other mental health outcomes at age 18 compared to their genetically identical cotwin, although the association with internalising symptoms at age 18 was no longer statistically significant after adjusting for prior emotional and behavioural problems and birth weight.

## Discussion

Consistent with our hypotheses, lower maternal warmth and higher maternal negativity at age 10 were associated with poorer mental health outcomes during adolescence. Many of these associations held even when taking into account the children's biological sex, family socioeconomic status and pre‐existing emotional and behavioural problems. Moreover, twins who experienced lower maternal warmth and higher maternal negativity than their cotwin generally experienced poorer mental health outcomes during adolescence than their cotwin. This is the first study to demonstrate that these associations extend into adolescence.

These findings extend previous research conducted in this cohort which used a similar methodology and suggested a potentially causal association between maternal EE at age 5 and antisocial behavioural problems at age 7 (Caspi et al., 2004). We show that such associations extend longer term, into adolescence, and to other mental health problems, including ADHD, conduct disorder and externalising symptoms, thought disorder symptoms and general psychopathology. We more consistently found associations between maternal negativity and adolescent mental health than for maternal warmth, suggesting that negativity may be particularly influential. Additionally, whereas some studies have shown that maternal EE is cross‐sectionally associated with childhood anxiety (Gar & Hudson, 2008) and depression (Schwartz et al., 1990), we more consistently found longitudinal associations with adolescent externalising problems than with internalising problems (including anxiety and depression) after adjusting for potential confounders. This finding is consistent with some previous research in younger age groups (Baker, Heller, & Henker, 2000; Peris & Baker, 2000). Possible explanations for this would be an important area for future research; for example, it may be that parental criticism may negatively impact offspring executive functions like inhibition (Blum & Ribner, 2022), which in turn could lead to more behavioural problems.

Our findings also suggest that some mothers in this cohort expressed quite different emotional attitudes towards their MZ twins. Some mothers of twins have been found to report having a preferred twin, or feeling like they understand or relate to one twin more than the other – this might explain some of the differential EE ratings identified among our MZ twins (Gowling, McKenzie‐McHarg, Gordon, & Harrison, 2021). Maternal mental illness, beliefs about twin dynamics, illness in one twin or associating one twin with an ex‐partner might also lead to some maternal EE discordance among twin offspring (Caspi et al., 2004). Moreover, these differences in emotional attitudes partly accounted for some of the differences subsequently seen in the MZ twins' adolescent mental health outcomes, especially those on the externalising spectrum. Importantly, focusing on twins who were brought up in the same household and are genetically identical in these twin‐difference analyses controls for potential unmeasured confounding in the shared family environment and genetic influences on these associations, which brings us a step closer to inferring that maternal EE is causally related to offspring mental health outcomes in adolescence.

However, it remains possible that other differences between twins could have resulted in differences in both maternal EE and in adolescent mental health outcomes, such as factors in the wider environment that were not shared between twins, like peer relationships (Plomin, 2011). Cognitive, biological and neurological differences between twins are another possibility. While in utero, it is not uncommon for one twin to receive more nutrition than the other, which can result in differences in body weight (Bagchi & Salihu, 2006) and regional brain volume (Wallace et al., 2006). The latter could lead to one twin having worse cognitive outcomes than the other twin (Peterson et al., 2000), which may in turn impact mothers' levels of EE towards them (Hastings & Lloyd, 2007). Poorer cognition in childhood may also put them at greater risk of developing mental health problems (Koenen et al., 2009). Low birth weight has also been associated with children displaying more behavioural problems (Gray, Edwards, Hughes, & Pritchard, 2018) which may result in poorer relationships with parents (Gralton, Doering, Ngui, Pan, & Schiffman, 2022) and greater likelihood of developing a range of mental health difficulties (Fitzallen, Taylor, & Bora, 2020). We adjusted for birth weight as a proxy for such neurological and behavioural differences in our twin‐difference analyses, and the associations remained; however, further, more detailed investigation of this potential confounding factor is required. Another possibility is that pre‐existing mental health issues in the children might affect both their mothers' EE (Bell & Harper, 1977) and increase their likelihood of experiencing mental health issues in adolescence (Oldehinkel & Ormel, 2023). In our analyses, we adjusted for twins' emotional and behavioural problems at age 5 (before the EE assessment took place) and found that the associations held, which provides reassurance that this reverse causality was not occurring. Nonetheless, we cannot fully rule this out, and a bidirectional relationship between maternal EE and offspring mental health remains possible.

### Implications

The findings from this study have the potential to inform the targeting and focus of preventive interventions. For example, a previous intervention study found that parent EE training (comprising psychoeducation about the role of EE in children's symptomatology, communication skills training and contingency management training) delivered alongside offspring cognitive behavioural therapy reduced adolescent mental health symptoms, particularly if the parents changed EE status (Garcia‐Lopez, Díaz‐Castela, Muela‐Martinez, & Espinosa‐Fernandez, 2014). While parenting interventions could helpfully target both warmth and negativity, our findings indicate that helping parents to manage negativity could be particularly influential for offspring mental health. For example, it might be beneficial to help parents develop strategies for situations where negativity towards their offspring is most likely to be elicited, like tackling challenging behaviours without making generalised comments about the child more broadly.

If future studies replicate and extend our findings to demonstrate that EE is a causal predictor of adolescent mental health, then high levels of negativity and low levels of warmth extracted from just five minutes of a parent talking about their child could indicate which families might benefit most from interventions to improve parent–child interactions. In turn, this could mitigate the onset of mental health problems in young people. However, the feasibility of using EE in practice (e.g. by social workers, health visitors or mental health teams that work with parents of small children) to identify high‐risk families is severely limited; training people to code speech samples for EE and conducting coding is very time‐consuming. We are therefore currently developing automated approaches to coding EE from speech, which would substantially speed up the process and increase the feasibility of assessing EE for future research and practice. Our preliminary work automating maternal warmth coding shows promise (Mirheidari et al., 2024), and further work is underway to automate maternal negativity coding, as well as consider the wider ethical, privacy and social implications of such approaches.

### Strengths and limitations

Strengths of this study include its longitudinal design and its use of the twin‐design, allowing us to investigate and rule out unmeasured shared environmental and genetic confounding in the associations under study. We also used validated measures for EE and mental health and considered both maternal warmth and maternal negativity, acknowledging that having high ratings on one does not necessarily imply low ratings on the other. A wide variety of other approaches have also been developed for eliciting and scoring EE, including different FMSS probes and scoring frameworks which capture more specific components such as criticism (Sher‐Censor, 2015); these could present areas for further exploration in future studies.

However, there are also a number of limitations. Generalisability of our findings may be limited, first beyond England and Wales (where the E‐Risk cohort were recruited from), second to various ethnic and cultural backgrounds (some of which are less well represented in the E‐Risk cohort) and third to nontwins, although twins and singletons score similarly on measures of emotional and behavioural problems (Moilanen et al., 1999). Another limitation is that we did not consider the role of some other, potentially important factors for the relationship under study. For example, parental mental illness has been found to be associated with higher EE and criticism (Fahrer, Brill, Dobener, Asbrand, & Christiansen, 2021). Our twin difference analysis accounts for shared environmental factors, but parental mental health could present an unmeasured confounder in our observed association if it is experienced differently by the twins. Indeed, if parental mental illness leads to discordant EE between twins, EE might be a mechanism for intergenerational transmission of mental health disorders. We also did not consider the role of paternal EE and mental health in this study – as compared to mothers, fathers and other caregivers are relatively understudied in relation to offspring mental health outcomes, despite research suggesting a potentially important role (Brennan, Hammen, Katz, & Le Brocque, 2002; Wickersham, Leightley, Archer, & Fear, 2020). It would therefore be important to consider the role of fathers in future EE research. School experiences could present further nonshared environmental risk factors which may play a role in the observed associations. While teacher‐reported externalising problems contributed to some of our outcome variables and covariates, therefore likely capturing their behaviours in the school environment to some degree, understanding the specific role of school experiences in more detail would warrant investigation in future studies.

## Conclusions

In this longitudinal twin study, we found evidence for an association between maternal EE at age 10 and adolescent mental health outcomes. Maternal negativity appeared to have a particularly consistent association with mental health measured at ages 12 and 18, especially symptoms on the externalising spectrum. Using a genetically sensitive design, we also showed that MZ twins were not identical in their maternal warmth and negativity ratings, and that discrepancies in the emotional attitudes expressed by mothers towards their MZ twins accounted for some of the subsequent discrepancies seen in their mental health outcomes during adolescence. This association cannot be attributed to genetic influences or the shared family environment, and is unlikely to be accounted for by neurological or prior emotional and behavioural differences between the twins, providing evidence for a potentially causal relationship. Maternal EE measurement could therefore offer an opportunity to identify families who might most benefit from family interventions, and children and adolescents who might benefit from mental health prevention programmes.

## Ethical considerations

The Joint South London and Maudsley and the Institute of Psychiatry Research Ethics Committee approved each phase of the study (NRES 1997/122). Parents gave informed consent and twins gave assent between 5 and 12 years and then gave informed consent at age 18.

**Key points**

- Maternal expressed emotion (EE) is associated with offspring's mental health in childhood, but there is little research exploring whether this continues into adolescence.
- In a longitudinal twin study, we found that maternal EE (specifically lower warmth and higher negativity) at age 10 was associated with worse mental health outcomes at ages 12 and 18.
- Moreover, discrepancies in maternal warmth/negativity ratings between monozygotic twins were linked to discrepancies in their externalising behaviours at age 12 and all forms of psychopathology at age 18, ruling out a genetic basis for these relationships and other forms of confounding in twins' shared environments.
- Maternal EE ratings might provide an opportunity to identify families who would benefit from family interventions and mental health prevention programmes.

## Supporting information


**Table S1.** STROBE Statement – Checklist of items that should be included in reports of cohort studies (Vandenbroucke et al., 2007).
**Appendix S1.** Additional details on maternal expressed emotion (EE) measurement, reproduced from Caspi et al. (2004).
**Appendix S2.** Additional details on measures of adolescent mental health at age 18, reproduced from Reuben et al. (2021).
**Figure S1.** The structure of psychopathology at age 18 years in the E‐Risk Cohort.
**Table S2.** Number and proportion of participants with missing data on each variable under study, total *n* = 2,232 at age 5.
**Table S3.** Characteristics of the full sample, and of individuals with complete data for both twins (excluding birth weight)

## Data Availability

The data that support the findings of this study are not publicly available but can be accessed with permission from the E‐Risk Study team: https://eriskstudy.com/data‐access/. A.W. and H.L.F. had full access to the study data. Supporting Stata code will become publicly available via A.W.'s GitHub account on publication: https://github.com/AliceWickersham.
