# Supplementary material for: Maternal expressions of warmth and negativity and adolescent mental health: using longitudinal monozygotic twin‐difference analyses to approach causal inference
Source: J Child Psychol Psychiatry. 2025 Aug 13;67(1):92–103. doi: 10.1111/jcpp.70020 (PMC12699114; doi:10.1111/jcpp.70020)
Supplement: Supplementary file 1 — Table S1. STROBE Statement – Checklist of items that should be included in reports of cohort studies (Vandenbroucke et al., 2007). Appendix S1. Additional details on maternal expressed emotion (EE) measurement, reproduced from Caspi et al. (2004). Appendix S2. Additional details on measures of adolescent mental health at age 18, reproduced from Reuben et al. (2021). Figure S1. The structure of psychopathology at age 18 years in the E‐Risk Cohort. Table S2. Number and proportion of participants with missing data on each variable under study, total n = 2,232 at age 5. Table S3. Characteristics of the full sample, and of individuals with complete data for both twins (excluding birth weight) [file JCPP-67-92-s001.docx]

**Supplementary Materials**

Wickersham *et al.* Maternal expressions of warmth and negativity and adolescent mental health: Using longitudinal monozygotic twin-difference analyses to approach causal inference.

*Table S1: STROBE Statement—Checklist of items that should be included in reports of cohort studies (Vandenbroucke et al., 2007)*

|  | Item No | Recommendation | Page No |
| --- | --- | --- | --- |
| **Title and abstract** | 1 | (*a*) Indicate the study’s design with a commonly used term in the title or the abstract | 2 |
|  |  | (*b*) Provide in the abstract an informative and balanced summary of what was done and what was found | 2 |
| Introduction | | | |
| Background/rationale | 2 | Explain the scientific background and rationale for the investigation being reported | 3-4 |
| Objectives | 3 | State specific objectives, including any prespecified hypotheses | 4 |
| Methods | | | |
| Study design | 4 | Present key elements of study design early in the paper | 2, 4 |
| Setting | 5 | Describe the setting, locations, and relevant dates, including periods of recruitment, exposure, follow-up, and data collection | 4-5 |
| Participants | 6 | (*a*) Give the eligibility criteria, and the sources and methods of selection of participants. Describe methods of follow-up | 4-5 |
|  |  | (*b*) For matched studies, give matching criteria and number of exposed and unexposed | N/A |
| Variables | 7 | Clearly define all outcomes, exposures, predictors, potential confounders, and effect modifiers. Give diagnostic criteria, if applicable | 6-8 |
| Data sources/ measurement | 8* | For each variable of interest, give sources of data and details of methods of assessment (measurement). Describe comparability of assessment methods if there is more than one group | 6-8 |
| Bias | 9 | Describe any efforts to address potential sources of bias | 5, 8 |
| Study size | 10 | Explain how the study size was arrived at | 5, 9 |
| Quantitative variables | 11 | Explain how quantitative variables were handled in the analyses. If applicable, describe which groupings were chosen and why | 8-9 |
| Statistical methods | 12 | (*a*) Describe all statistical methods, including those used to control for confounding | 8-9 |
|  |  | (*b*) Describe any methods used to examine subgroups and interactions | 8-9 |
|  |  | (*c*) Explain how missing data were addressed | 9 |
|  |  | (*d*) If applicable, explain how loss to follow-up was addressed | 5, 9 |
|  |  | (*e*) Describe any sensitivity analyses | 8, 9 |
| Results | | |  |
| Participants | 13* | (a) Report numbers of individuals at each stage of study—eg numbers potentially eligible, examined for eligibility, confirmed eligible, included in the study, completing follow-up, and analysed | 5, 9, S3 |
|  |  | (b) Give reasons for non-participation at each stage | 5, 6 |
|  |  | (c) Consider use of a flow diagram | - |
| Descriptive data | 14* | (a) Give characteristics of study participants (eg demographic, clinical, social) and information on exposures and potential confounders | 9, 22-23, |
|  |  | (b) Indicate number of participants with missing data for each variable of interest | 9, S2 |
|  |  | (c) Summarise follow-up time (eg, average and total amount) | 5 |
| Outcome data | 15* | Report numbers of outcome events or summary measures over time | 9, 23 |
| Main results | 16 | (*a*) Give unadjusted estimates and, if applicable, confounder-adjusted estimates and their precision (eg, 95% confidence interval). Make clear which confounders were adjusted for and why they were included | 9- 10, 24-25 |
|  |  | (*b*) Report category boundaries when continuous variables were categorized | N/A |
|  |  | (*c*) If relevant, consider translating estimates of relative risk into absolute risk for a meaningful time period | N/A |
| Other analyses | 17 | Report other analyses done—eg analyses of subgroups and interactions, and sensitivity analyses | 10, 26-28 |
| **Discussion** |  |  |  |
| Key results | 18 | Summarise key results with reference to study objectives | 11 |
| Limitations | 19 | Discuss limitations of the study, taking into account sources of potential bias or imprecision. Discuss both direction and magnitude of any potential bias | 13-14 |
| Interpretation | 20 | Give a cautious overall interpretation of results considering objectives, limitations, multiplicity of analyses, results from similar studies, and other relevant evidence | 14 |
| Generalisability | 21 | Discuss the generalisability (external validity) of the study results | 13 |
| **Other information** |  |  |  |
| Funding | 22 | Give the source of funding and the role of the funders for the present study and, if applicable, for the original study on which the present article is based | 15 |

*Give information separately for exposed and unexposed groups.

*Appendix S1: Additional details on maternal expressed emotion (EE) measurement, reproduced from (Caspi et al., 2004)*

The measurement of EE in developmental psychopathology is distinguished by four key features: (a) It focuses on individual-specific EE (i.e., individual with respect to both the person expressing the emotion and the child receiving it); (b) it refers to emotions observed in the manner in which an adult talks about a child, rather than by answers to specific closed-ended questions; (c) it uses both verbal and vocal elements in rating emotions (that is, both what is said and the tone of voice used); and (d) it focuses on emotions about the child as an individual, rather than on emotions concerned with a child’s symptoms.

The E-Risk Study uses a novel approach to scoring EE given concerns that have been raised about the developmental inappropriateness of the standard scoring protocol originally developed for studies of adult psychiatric patients (e.g., Daley, Sonuga-Barke, & Thompson, 2003; McCarty & Weisz, 2002; Sandberg, Rutter, & Järvi, 2003).

***Coding of maternal warmth***

The scale refers only to the warmth expressed in the interview about the child. The warmth of the respondent’s personality was not a consideration, nor was warmth shown toward others. Positive comments in themselves were not viewed as evidence of warmth, nor were stereotyped endearments. Warmth was assessed by the tone of voice, spontaneity (e.g., ‘She is so funny—the other day she made up a song and she was dancing and singing in the garden . . . the song was about everything . . . a butterfly flew by and that ended up in the song . . . it was so sweet.’), sympathy and/or empathy toward the child (e.g., ‘I feel really sorry for her, it is not her fault . . . I worry for her.’).

Warmth was coded on a 6-point scale. *High warmth* (5) and *moderately high warmth* (4) were coded when there was definite and clear-cut tonal warmth, enthusiasm, interest in, and enjoyment of the child. For example, ‘She is a delight, she is so happy, I love taking her out, she is my ray of sunshine’ was coded as a 5. *Moderate warmth* (3) was coded when there was definite understanding, sympathy, and concern but only limited warmth of tone, for example, ‘I worried about her when she went to school, I thought she may have difficulty in mixing, and I felt sorry for her.’ *Some warmth* (2) was coded when the mother showed a detached, rather clinical approach and little or no warmth of tone, but moderate understanding, sympathy, and concern. For example, an interview with comments along the lines of ‘She’s alright’ with little substantiation would have received this rating*. Very little warmth* (1) was rated when there was only a slight amount of understanding, sympathy, concern, enthusiasm about, or interest in the child*. No warmth* (0) was reserved for mothers who showed a complete absence of the qualities of warmth as defined.

***Coding of maternal negativity***

As with warmth, a 6-point rating scale was used, referring to the negativity expressed in the interview about the child. *No negativity* (0) was coded when the mother made no negative comments about the child*. A little negativity* (1) was coded when the mother made one minor criticism such as ‘She is lazy.’ *Some negativity* (2) was coded when the mother made two criticisms that were stronger in tone than the former rating. The next three codes were considered present when maternal negativity was generalised to the child himself or herself rather than against particular behaviours or attributes. These ratings were used when the tone and content of the interview were primarily negative. *Negative—some dissatisfaction* (3) was coded when the mother repeatedly mentioned one or two particular traits of the child that she did not like and wished to change, for example, ‘She is not very clever; it would help if she tried more, but she doesn’t; I wish she would try more, like her sister.’ This was the general theme of this particular EE interview with the mother, and it was thus rated a 3. *Negative—makes disparaging remarks and finds fault with the child* (4) was coded when the mother had very little good to say about her child and found fault in almost everything he or she did, for example, ‘She always does it; I have never met such a clumsy child; we think ‘Oh here we go again, she’s done it again’; it drives me mad; why doesn’t she look where she is going? I’m constantly having to look out for her; she’s constantly breaking things . . . sometimes I think she is stupid, she never learns.’ *Resentful and hostile* (5) was coded when the mother gave the impression that she actively disliked the child. The interview would take the form of a stream of negativity against the child, with no positive comments, for example, ‘I wish I had never had her . . . she’s a cow, I hate her’.

*Appendix S2: Additional details on measures of adolescent mental health at age 18, reproduced from (Reuben et al., 2021)*

*Assessment of symptoms of mental disorder*

At age 18, participants were assessed in private interviews about past-year symptoms of mental disorders (Schaefer et al., 2018). Five externalizing-spectrum disorder symptoms were assessed: Diagnostic and Statistical Manual of Mental Disorders 4th edition (DSM–IV) (American Psychiatric Association, 1994) symptoms of alcohol dependence and cannabis dependence assessed via the Diagnostic Interview Schedule (DIS) (Robins, Cottler, Bucholz, & Compton, 1995); conduct disorder assessed by inquiring about DSM–IV symptoms; symptoms of tobacco dependence assessed via the Fagerstrom Test for Nicotine Dependence (Heatherton, Kozlowski, Frecker, & FAGERSTROM, 1991); and attention-deficit/ hyperactivity disorder (ADHD) assessed by inquiring about DSM 5th edition (DSM–5) symptoms (Agnew-Blais et al., 2016; American Psychiatric Association, 2013). Four internalizing-spectrum disorder symptoms were assessed: DSM–IV symptoms of depression, generalized anxiety disorder, and posttraumatic stress disorder (PTSD) assessed via the DIS (Robins et al., 1995), and symptoms of eating disorder assessed via the SCOFF (Morgan, Reid, & Lacey, 1999). Thought disorder symptoms were assessed in two ways: first, participants were asked 7 items about delusions and hallucinations (psychotic-like experiences: e.g., “Have other people ever read your thoughts?”; “Have you ever thought you were being followed or spied on?”; “Have you ever heard voices that other people cannot hear?”) (Polanczyk et al., 2010). Second, participants were asked 6 items about unusual thoughts and feelings (prodromal symptoms: e.g., “My thinking is unusual or frightening”; “People or places I know seem different”), drawing on item pools since formalized in prodromal psychosis instruments, including the PRIME-screen and SIPS (Loewy, Pearson, Vinogradov, Bearden, & Cannon, 2011).

*The structure of psychopathology*

Using confirmatory factor analysis, two standard models (Brunner, Nagy, & Wilhelm, 2012; Rindskopf & Rose, 1988) that are frequently used to examine hierarchically structured constructs were estimated: a correlated-factors model with three factors (representing Internalizing, Externalizing, and Thought Disorder symptoms) and a bi-factor model specifying a General Psychopathology factor (Figure S1) in addition to the three specific factors. Decisions about symptom-factor loadings were guided by the Hierarchical Taxonomy of Psychopathology consortium (<https://medicine.stonybrookmedicine.edu/HITOP/AboutHiTOP>) (Kotov et al., 2017). Symptoms corresponding to disorders of distress (depression, generalized anxiety disorder, and PTSD) and eating pathology loaded on the Internalizing factor; symptoms corresponding to disorders of substance use (alcohol, cannabis, tobacco) and oppositional behaviour (conduct disorder) and ADHD loaded on the Externalizing factor; and symptoms corresponding to disorders associated with psychosis loaded on the Thought Disorder factor. Confirmatory factor analyses were run as two-level clustered models to account for the nesting of twins within families, with analyses performed in MPlus v7.4 (Muthén & Muthén, 2017) using the robust maximum likelihood estimator (MLR) to provide standard errors that are robust to non-normality and non-independence of observations.

Both models fit the data well as assessed by the Akaike Information Criterion (AIC), Bayesian Information Criterion (BIC) and the Sample Adjusted BIC, although the bi-factor model demonstrated marginally superior fit.

For the correlated-factors model, AIC=42987.116, BIC=43488.486, Sample Adjusted BIC=43205.726. Loadings on each of the three factors were all positive, generally high (all p’s < .001) and averaged 0.680 (Externalizing: average loading=0.638; Internalizing: average loading=0.654; Thought Disorder: average loading=0.836). Correlations between the three factors were all positive and ranged from 0.552 between Externalizing and Thought Disorder to 0.756 between Internalizing and Thought Disorder. Thus, this model confirmed that three correlated factors (i.e., Internalizing, Externalizing, and Thought Disorder) explained the structure of the 11 symptom scales examined in the E-Risk twins at age 18.

For the bi-factor model, AIC=42897.350, BIC=43443.787, Sample Adjusted BIC=43135.609. Loadings on the General Psychopathology factor (“p”) were all positive, generally high (all p’s < .001) and averaged 0.519; the highest standardized loadings were for psychotic symptoms (0.759 and 0.592), major depressive episode (0.718), eating disorders (0.574), and generalized anxiety disorder (0.567). Similarly, the loadings for the three style factors were all positive and averaged 0.507 for Externalizing, 0.270 for Internalizing, and 0.496 for Thought Disorder. Thus, this model confirmed that a bi-factor structure (i.e., with a General Psychopathology factor and three specific Internalizing, Externalizing, and Thought Disorder factors) explained the structure of the 11 symptom scales examined in the E-Risk twins at age 18.

***Figure S1: The structure of psychopathology at age 18 years in the E-Risk Cohort.***


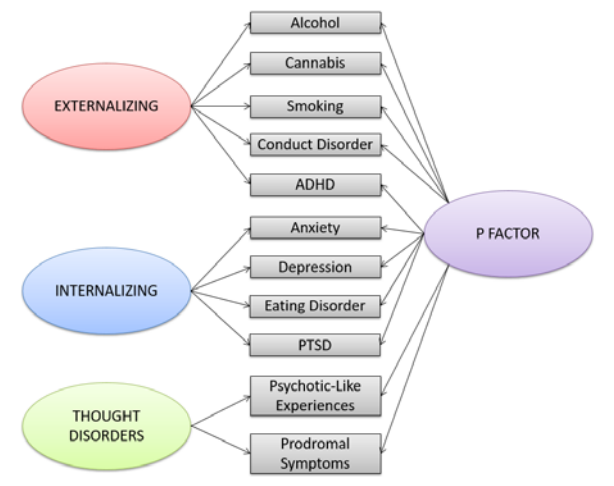
**(**A)

**(**B)


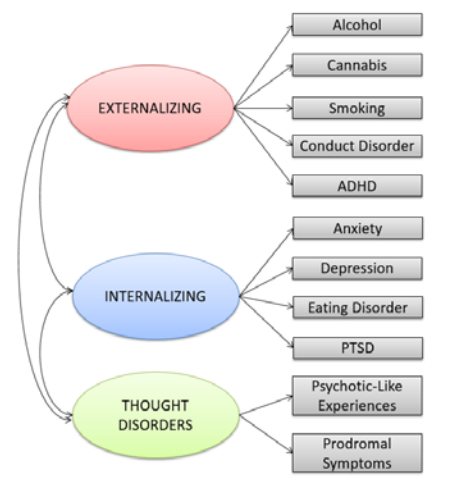


*Note.* (A) Bi-factor model, (B) Correlated-factors model. Coloured ovals represent latent (unobserved) continuous symptom trait factors; grey boxes represent age-18 observed scores on symptom scales corresponding to each disorder. ADHD = attention-deficit/hyperactivity disorder, PTSD = post-traumatic stress disorder. P-Factor represents the factor of General Psychopathology. Figure reproduced from Reuben et al. (2021) and Schaefer et al. (2018).

*Table S2: Number and proportion of participants with missing data on each variable under study, total n=2,232 at age 5*

| **Variable** | **Missing observations, n (%)** |
| --- | --- |
| Birth weight | 156 (7.0) |
| Maternal warmth (age 10) | 132 (5.9) |
| Maternal dissatisfaction/negativity (age 10) | 131 (5.9) |
| Depression scale (age 12) | 102 (4.6) |
| Anxiety scale (age 12) | 102 (4.6) |
| ADHD scale (age 12) | 91 (4.1) |
| Conduct disorder scale (age 12) | 87 (3.9) |
| General psychopathology [P-factor] (age 18) | 166 (7.4) |
| Internalising symptoms (age 18) | 166 (7.4) |
| Externalising symptoms (age 18) | 166 (7.4) |
| Thought disorder symptoms (age 18) | 166 (7.4) |

*Note: All remaining variables included in analyses had no missing data.*

*Abbreviations: ADHD = attention deficit hyperactivity disorder.*

*Table S3: Characteristics of the full sample, and of individuals with complete data for both twins (excluding birth weight)*

|  | **Full sample**  **(n=2,232)** | **Complete cases**  **(n=1,906)** |
| --- | --- | --- |
|  | **Frequency (%)** | |
| **Biological sex at birth (n=2,232)** |  |  |
| *Male* | 1,092 (48.9) | 892 (46.8) |
| *Female* | 1,140 (51.1) | 1,014 (53.2) |
| **Zygosity (n=2,232)** |  |  |
| *Monozygotic* | 1,242 (55.7) | 1,058 (55.5) |
| *Dizygotic* | 990 (44.4) | 848 (44.5) |
| **Family socioeconomic status, age 5 (n=2,232)** |  |  |
| *Low* | 742 (33.2) | 636 (33.4) |
| *Medium* | 738 (33.1) | 622 (32.6) |
| *High* | 752 (33.7) | 648 (34.0) |
| **Ethnicity (n=2,232)** |  |  |
| *White* | 2,018 (90.4) | 1,724 (90.5) |
| *Asian* | 90 (4.0) | 78 (4.1) |
| *Black* | 42 (1.9) | 34 (1.8) |
| *Mixed race* | 8 (0.4) | 6 (0.3) |
| *Other* | 74 (3.3) | 64 (3.4) |
|  | **Mean (SD)** | |
| **Birth weight (n=2,076)** | 2438.3 (539.9) | 2446.7 (536.2) |
| **Maternal expressed emotion, age 10** |  |  |
| *Warmth (n=2,100)* | 3.7 (0.9) | 3.7 (0.9) |
| *Dissatisfaction/negativity (n=2,101)* | 1.4 (0.9) | 1.4 (0.9) |
| **Mental health, age 5** |  |  |
| *Behavioural problems (n=2,232)* | 18.3 (13.7) | 18.0 (13.7) |
| *Emotional problems (n=2,232)* | 12.1 (8.3) | 12.0 (8.1) |
| **Mental health, age 12** |  |  |
| *Depression symptoms (n=2,130)* | 3.1 (5.3) | 3.1 (5.3) |
| *Anxiety symptoms (n=2,130)* | 7.6 (3.0) | 7.6 (3.0) |
| *ADHD symptoms (n=2,141)* | 12.3 (11.1) | 12.1 (11.1) |
| *Conduct disorder symptoms (n=2,145)* | 1.0 (1.7) | 1.0 (1.7) |
| **Mental health, age 18** |  |  |
| *General psychopathology [P-factor] (n=2,066)* | 100.0 (15.0) | 99.8 (15.0) |
| *Internalising symptoms (n=2,066)* | 100.0 (15.0) | 99.8 (15.0) |
| *Externalising symptoms (n=2,066)* | 100.0 (15.0) | 99.7 (15.0) |
| *Thought disorder symptoms (n=2,066)* | 100.0 (15.0) | 99.8 (14.9) |

*Abbreviations: ADHD = attention deficit hyperactivity disorder; SD = Standard Deviation.*

**References**

Agnew-Blais, J. C., Polanczyk, G. V., Danese, A., Wertz, J., Moffitt, T. E., & Arseneault, L. (2016). Evaluation of the persistence, remission, and emergence of attention-deficit/hyperactivity disorder in young adulthood. *JAMA Psychiatry, 73*(7), 713-720.

American Psychiatric Association. (1994). *Diagnostic and Statistical Manual of Mental Disorders, 4th edition*. Washington, DC: American Psychiatric Association.

American Psychiatric Association. (2013). *Diagnostic and Statistical Manual of Mental Disorders, 5th edition*. Washington, DC: American Psychiatric Association.

Brunner, M., Nagy, G., & Wilhelm, O. (2012). A tutorial on hierarchically structured constructs. *Journal of Personality, 80*(4), 796-846.

Caspi, A., Moffitt, T. E., Morgan, J., Rutter, M., Taylor, A., Arseneault, L., . . . Polo-Tomas, M. (2004). Maternal expressed emotion predicts children's antisocial behavior problems: Using monozygotic-twin differences to identify environmental effects on behavioral development. *Developmental Psychology, 40*(2), 149-161.

Daley, D., Sonuga-Barke, E. J., & Thompson, M. (2003). Assessing expressed emotion in mothers of preschool AD/HD children: Psychometric properties of a modified speech sample. *British Journal of Clinical Psychology, 42*(1), 53-67.

Heatherton, T. F., Kozlowski, L. T., Frecker, R. C., & Fagerstrom, K. O. (1991). The Fagerström test for nicotine dependence: a revision of the Fagerstrom Tolerance Questionnaire. *British Journal of Addiction, 86*(9), 1119-1127.

Kotov, R., Krueger, R. F., Watson, D., Achenbach, T. M., Althoff, R. R., Bagby, R. M., . . . Clark, L. A. (2017). The Hierarchical Taxonomy of Psychopathology (HiTOP): A dimensional alternative to traditional nosologies. *Journal of Abnormal Psychology, 126*(4), 454.

Loewy, R. L., Pearson, R., Vinogradov, S., Bearden, C. E., & Cannon, T. D. (2011). Psychosis risk screening with the Prodromal Questionnaire—brief version (PQ-B). *Schizophrenia Research, 129*(1), 42-46.

McCarty, C. A., & Weisz, J. R. (2002). Correlates of expressed emotion in mothers of clinically‐referred youth: An examination of the five‐minute speech sample. *Journal of Child Psychology and Psychiatry, 43*(6), 759-768.

Morgan, J. F., Reid, F., & Lacey, J. H. (1999). The SCOFF questionnaire: assessment of a new screening tool for eating disorders. *BMJ, 319*(7223), 1467-1468.

Muthén, L., & Muthén, B. (2017). *MPlus User’s Guide, 8th edition*. Los Angeles, CA: Muthén & Muthén.

Polanczyk, G., Moffitt, T. E., Arseneault, L., Cannon, M., Ambler, A., Keefe, R. S., . . . Caspi, A. (2010). Etiological and clinical features of childhood psychotic symptoms: results from a birth cohort. *Archives of General Psychiatry, 67*(4), 328-338.

Reuben, A., Arseneault, L., Beddows, A., Beevers, S. D., Moffitt, T. E., Ambler, A., . . . Schaefer, J. D. (2021). Association of air pollution exposure in childhood and adolescence with psychopathology at the transition to adulthood. *JAMA Network Open, 4*(4), e217508-e217508.

Rindskopf, D., & Rose, T. (1988). Some theory and applications of confirmatory second-order factor analysis. *Multivariate Behavioral Research, 23*(1), 51-67.

Robins, L., Cottler, L., Bucholz, K., & Compton, W. (1995). *Diagnostic Interview Schedule for DSM-IV*. St. Louis: Washington University School of Medicine.

Sandberg, S., Rutter, M., & Järvi, J. (2003). Brief measure of expressed emotion: Internal consistency and stability over time. *International Journal of Methods in Psychiatric Research, 12*(4), 182-191.

Schaefer, J. D., Moffitt, T. E., Arseneault, L., Danese, A., Fisher, H. L., Houts, R., . . . Caspi, A. (2018). Adolescent victimization and early-adult psychopathology: Approaching causal inference using a longitudinal twin study to rule out noncausal explanations. *Clinical Psychological Science, 6*(3), 352-371.

Vandenbroucke, J. P., Elm, E. v., Altman, D. G., Gøtzsche, P. C., Mulrow, C. D., Pocock, S. J., . . . Initiative, S. (2007). Strengthening the Reporting of Observational Studies in Epidemiology (STROBE): Explanation and elaboration. *Annals of Internal Medicine, 147*(8), W-163-W-194.
